# Religious Identity Construction in Transnational Family Talks: An Autoethnographic Study

**DOI:** 10.3390/children10020277

**Published:** 2023-01-31

**Authors:** Muhammad Alasmari

**Affiliations:** Department of English, College of Sciences & Arts and the Applied College at Al-Namas, Univeristy of Bisha, Bisha 67389, Saudi Arabia; moaalasmri@ub.edu.sa

**Keywords:** bilingualism, family language policy, multilingualism, religious identity

## Abstract

Studies in Family Language Policy (FLP) have focused more on language patterns and practices in transnational families with little attention to the challenges of multilingualism. Through exploring diverse experiences in dealing with multilingualism, more can be understood about parent language ideology, the enactment of FLP, and factors related to identity construction. Therefore, the study highlights how the family experiences affect the way individual family members perceive social relations and social structures and how they construct and present their own identities. The study is based on an analysis of longitudinal data from children’s family transnational experiences and how the dynamic of FLP affected not only family talk style but also identity construction. The main focus of the study entails an analysis of personal auto-ethnographic accounts. The study explored the emergence of religious identity in the family talks via (1) using referring expressions to talk about religious places related to two different contexts, and (2) frequently using religious phrases in different settings, which, in turn, reveal the interplay between macro and micro factors affecting the parental language ideology, language planning, and identity construction in the FLP. This study contributed to the field of language policy by presenting the different trajectories in identity construction and family language in a transnational families from a less explored religious and ethnic group.

## 1. Introduction

Researchers have explored language use among adults and children in the home context through cognitive, educational, social, and cultural lenses. Such perspectives complement each other as they give a clearer picture of the language acquisition process and interaction among family members. Earlier studies concentrated more on how to raise a bilingual child [1], with later studies discussing positive and negative aspects of bilingualism [2], classifying types of home input and bilingualism [3], examining grammatical or phonological trajectories among children [4], and looking at sociocultural factors [5]. Together, this research has provided a deeper understating of bilingual or multilingual language acquisition and behavior in the home, albeit mostly from psychological and language acquisition perspectives.

Over the last decade, researchers have started investigating language use at home and its implications from different perspectives, as issues related to interactional strategies and social life within the family have drawn increasing attention from scholars [6,7]. Therefore, FLP, as an emerging field of inquiry, has derived from and contributed to studies related to language policy and child language acquisition [8] with more focus on investigating language policy in families and individuals in home domains. FLP research has been dedicated to understanding different dimensions, such as a parent’s role and aspirations in developing multilingualism in the family, external socio-political and linguistic factors impacting interaction among family members in the home [8], and parental discourse strategies regarding input and the linguistic environment in raising bilingual children [9].

This body of FLP research has revealed broad economic and educational factors shaping parents’ language ideologies and practices at home. Studies have highlighted the conflict between parents’ language ideologies and aspirations to maintain language policy in different family contexts. Objectives have ranged from finding the link between ideology and practice to investigate what external factors impact parents’ ideologies and practices. It is necessary to note the intersection between these external factors and other contextual factors in language ideology. Furthermore, more discussion is needed on how religion and religious institutions affect Muslim language ideologies as other domains of life involving the workplace, religious institutions, and recreation and their relation to FLP need yet to be explored [10]. 

The research article consists of six main sections. Section 1 introduces the study. Section 2 gives a detailed theoretical background on factors impacting parental language ideology, parents’ ideologies and language use at home, and parental language ideologies and family religious identity. Section 3 gives hints on methodology and materials including research design, the participants and selection criteria, and data collection and data analysis. Section 4 presents the results of the study. Section 5 includes a discussion of the study with reference to other studies, and Section 6 gives a summary of the findings of the study.

## 2. Theoretical Background

### 2.1. Factors Impacting Parental Language Ideology

FLP studies have ranged from demographic and material to more conceptual and theoretical [11] and from studying parents’ attitudes, beliefs, ideologies, and practices to children’s perspectives and agentive roles [12]. Recent studies have shifted to focus more on child and adult language use to understand the “meaning-making and the language-mediated experiences of multilingual families” and the interplay between language ideologies and practices [12]. However, there is a lack of studies exploring family and individual experiences dealing with multilingualism. Hua and Wei [13] clearly summarized the research gap.

In research, bilingualism and multilingualism need to be studied as experience, and experiences need to be studied holistically and multi-dimensionally. Identifying overall patterns and analyzing the details of interactional episodes are useful and necessary. But they need to be contextualized within the broader experiences of the individuals, families, and communities concerned. Whilst we celebrate the benefits of bilingualism and multilingualism, we should avoid romanticizing them, or seeing them as universally positive experiences. Bilingualism and multilingualism are a reality in contemporary society. They are also a challenge to us all.

One topic in FLP is explaining factors influencing parents’ ideologies, decisions, planning, and management. Curdt-Christiansen [14] examined language ideologies in light of how language was maintained, practiced, valued, and connected to certain linguistic patterns in a given context. Through ethnographic inquiry tools, she found that socio-political and economic factors influenced the FLP. More specifically, parents’ level of education, immigration experience, and cultural practices contributed to their expectations in the FLP. In addition, the findings suggested that socio-political and economic factors related to minority and majority contexts affected parental ideologies and impacted parental expectations and aspirations and as a result the family language policies.

In a later study, FLP was developed within transnational bilingual families in Singapore with respect to their children learning Chinese. She focused on parents’ perceptions of Chinese and the correlations between their beliefs and language practices. All parents had explicit beliefs in the benefits of Chinese language development for cultural identity and future socioeconomic opportunities. In addition, factors such as national language policy and language values shaped language policy and practice at the family level. This could explain the conflict between parental ideologies and language practices [14].

This conflict is not limited to the language’s value, power distribution, and parents’ education level or immigration experience. Hua [15] found that conflict also existed because of sociocultural values and the way second and third generations perceived a language’s cultural associations. Using qualitative methods, she found that each Chinese mother and young child manipulated their linguistic repertoire to serve their sociocultural perspectives. The above studies shed light on transnational family experiences and efforts to cope with socioeconomic and political factors contributing to the ideologies, beliefs, and practices in family interaction, as well as which factors were reflective of practices.

### 2.2. Parents Ideologies and Language Use at Home

Other FLP studies have investigated the correlation or conflict between parents’ ideologies, decisions, and practices and the consistency of the home language. Schwartz [16] discussed the complexity of parent attitudes by examining factors affecting home language maintenance among second-generation Russian Jewish immigrants in Israel. The findings indicated a conflict between immigrant parents’ ideologies about L1 maintenance and reported practice. For example, although most parents showed positive attitudes toward the L1, only about a fourth tended to use it in literacy practices. Furthermore, children’s reports about their attitudes were more reflective of actual practice. In a different context, Lao [17] examined English–Chinese parents who enrolled their children in bilingual preschool programs to learn about their attitudes toward both languages, bilingual education, and their language use at home. A gap was found between parents’ expectations and actual practice, as parents’ expectations of their children’s language competence may have varied due to factors such as parents’ Chinese proficiency and use of the L1 at home.

Recently, Curdt-Christiansen [18] studied the parental ideologies behind language choice at home, focusing on what families did and did not do in their daily interactions. After collecting and analyzing about 700 min of interactions, the data vividly revealed conflicting ideologies and contradictions between ideologies and practice. The author highlighted the role of external as well as internal factors, such as discourse strategies, in shaping and linking ideologies to practice. In another study, Soler and Zabrodskaja [19] reported practices and related ideologies in three Spanish-Estonian families and their children. Through sociolinguistic interviews with family members, the researchers illustrated how parents’ ideologies, reported practices, and the new speaker agencies interacted. Parents had positive attitudes toward the “one parent, one language” (OPOL) strategy but reported frequently using language mixing techniques (translanguaging).

### 2.3. Parental Language Ideologies and Family Religious Identity 

FLP studies have examined how affiliation affected language policy and interactional processes. For example, Haque [20] investigated how linguistic repertoire supplemented religion’s influence on identity recognition among Indian immigrant families in four European countries. Considering the religious language influenced the identity of the parents, this study showed their impact on participants’ language practices and how it pushed young adults to show signs of identity stress. Yazan and Ali [21] examined how religion shaped parents’ language planning and linguistic decisions regarding their daughter’s Arabic maintenance. Interviews with parents in an Arab Libyan family in the U.S. showed religious identity as an important part of parents’ language ideologies and ethnolinguistic aspiration. Parents pushed for more identity construction in the family through immersion in Muslim communities and participating in religious practices. These studies approached religious affiliation as an external factor in language ideology. 

### 2.4. Research Question and Hypotheses

The FLP literature contains several gaps. First, researchers initially put greater effort into investigating language use and practices in certain types of families, in certain communities, and among limited socioeconomic classes [11,22]. Regarding the communities covered in FLP literature, Smith-Christmas [23] pointed out three main prototypical contexts, namely the “one parent, one language” (OPOL) context, immigrant communities, and autochthonous (indigenous) communities. This classification was based on language use at home (OPOL) or on the family’s socioeconomic status within an immigrant or autochthonous community. Furthermore, Smith-Christmas [24] called for researchers to include different family contexts, as the bulk of the family language literature is situated within families from a Western industrial nation relocated in another Western industrial nation with a Western minority language, such as German parents in Australia or American parents in Norway. The reason to include a non-Western industrial community when studying FLP is the impossibility of generalizing a Western privileged sociolinguistic perspective to other communities or parts of the world where linguistic, educational, and economic factors vary. Another factor is that the nature of childrearing and orientations toward the child’s agentive roles could vary from one community to another [24]. This study seeks to answer the following research question: How is religious identity constructed and negotiated in the process of bilingual family language socialization? The researcher sought to address this gap by investigating the FLP of my transnational family, reflecting on our bilingual parenting experience, and discussing the processes of religious identity construction and negotiation. The present study examined the dynamicity of a Muslim FLP during language socialization and times of mobility to present how religious identity is constructed and negotiated and to further our understanding of the ways economic, cultural, and educational factors affected a Muslim family’s language socialization and policy.

## 3. Materials and Methods

### 3.1. Research Design

In auto-ethnography, the researcher plays the role of researcher and participant at the same time; my role in this study was a father and linguist who aimed to provide a close look into identity construction and negotiation in family talks. I choose to study my own family experience with transnationalism and language socialization to reflect in our identity construction because we are coming from a demographic group that has been little explored in the literature [21]. Moreover, this study provides a comprehensive analysis of the experience of raising a bilingual child from a sociolinguistic ethnographic perspective. 

### 3.2. The Participants and Selection Criteria 

The current study participants are (1) Muhammad, the father and the researcher, a bilingual speaker; (2) Salma, the mother and an English language learner who enrolled in an intensive English program during the time of conducting this study; and (3) a 3.5-year-old girl, Juman, who was enrolled in an English pre-school program in the host country. The participants are sequential bilinguals who acquired Arabic from birth and English when they were older than 6 years. The setting of the study was mainly where normal interactions occur among the family members, such as at home, in the car, and, parks, and while traveling. 

I engaged in this study by controlling inseparable roles as both father and researcher by documenting, analyzing, and reflecting on my family’s experiences through an autoethnographic approach. Researchers who use an ethnographic approach usually employ a longitudinal participant observation method to obtain extensive and in-depth data [25]. Informal participant observation used to collect data, and audio recording used to help track the language practices and socialization in the target family.

Context of the Study:

The fieldwork took place at two different sites. Most fieldwork was collected and analyzed based on the bilingual context of the Memphis area in the U.S., where my family lived as an immigrant transnational family for educational purposes. In this site, we lived in a neighborhood where we were the only international family. The closest Saudi family was a 15-minute drive away. Similarly, the closest mosque was about 10 minutes away and included Muslim families from different cultural backgrounds. Also, during the time of this study, the child was enrolled in an English-only childcare center while the parents were using both languages at home.

The second site was where my family and I spent the summer in Saudi Arabia, a heterogenous place where Arabic was the national language and was ubiquitous around us. Since we visited Saudi Arabia for about 3 months, some of the data were collected based on this period of time. However, our status as a transnational mobile family continued to be relevant in this context. The consideration of our immigration history and the imagination of our future together influenced both research sites in way such as the goal of maintaining the Arabic language and using English with the child while visiting our home country.

### 3.3. Data Collection Procedure

Data in this paper was obtained from a larger data set of a main longitudinal study that looked into language socialization processes in an Arabic Muslim family living temporally in the west for 3 years. This study was based on autoethnographic approach to track religious identity construction in family talks while the family mobility between cultures, countries, and languages. Autoethnography guides this study as it is a research tradition that was founded in ethnography, and defined by Savin-Baden and Major [20] as the study of people, culture, and values. It is an approach that aims to build an understanding of those being studied. In applied linguistics, ethnography was introduced as the study of people’s behavior in naturally occurring, ongoing settings, with a focus on the cultural interpretation of behavior [21,26]. This approach is often used in qualitative applied linguistics research, which tends to study an individual or group of people and their culture to have a more complete understanding of participants’ experiences and how their life circumstances impact their interactions and reactions to their situations. Heigham and Crocker [27] stated that there are two reasons to consider using ethnography in applied linguistics: (1) it relies on recording behavior as it happens rather than relying on people’s recollection of the past or their ideas about the future, and (2) it provides the possibility of presenting the collected data in many different ways to reach a wide audience. 

### 3.4. Data Analysis Procedure

The data analysis included in this current study was obtained from the field notes of author observations, reflexive journals, and recorded natural conversations (total of 80 h) between family members that was qualitatively analyzed to gain a broader, multifaceted perspective. I restricted the autoethnographic writing process and data analysis to 2 years, from mid 2016 to the end of the summer of 2018, because in this time frame, my family and I faced major challenges in child rearing as our first and only child started Due to the amount and variation of data, I employed two qualitative analytical methods: analytical induction and discourse analysis. They were chosen for their ability to provide analytical lenses for studying personal experiences and social interactions.

## 4. Results

### 4.1. Pro-Bilingual FLP and Emergence of Religious Identity in Family Talk

Identity construction through language use in different developmental stages activates the individual’s social roles [28] and helps establish relationships of power and status between members of a community (or family) during communication. Two linguistic patterns were apparent in the data that contributed to identity construction during our pro-bilingual FLP. This result section discusses Juman’s identity construction in her new educational context, the English-only childcare center, and how my wife and I witnessed new trajectories in raising a bilingual child. Furthermore, the culture, values, and discourse of the new educational setting differed from the home. The mother and the father did not explicitly initiate or discuss the use of cultural-related phrases, as we were socialized to use them before marriage and continued using them because they were part of who we were. However, Juman started actively acquiring, using, and negotiating these phrases in the late stage of collecting data, when she was 3 and was attending the university childcare center. Furthermore, during this time, both languages (English and Arabic) were used at home between the family members. Following is a discussion of these strategies, how our parental conversations with Juman included linguistic elements that constructed our family’s religious identity, and how Juman perceived and participated in this identity construction during our stay in a bilingual context. This data presentation below reveals the interplay between macro and micro factors affecting our parental language ideology, language planning, and identity construction in our FLP.

### 4.2. Using Referring Expressions for Religious Places in Saudi and Host Contexts

The use of religious expressions in family talks went beyond instructional purposes. Such expressions served to achieve social and interactional goals and helped in the construction and negotiation of our family’s religious identity. Throughout this pro-bilingual FLP, family members used different proper nouns for places of worship in the home and host contexts. The times these phrases were mentioned in family conversations were tracked and are presented in Table 1.

All terms in Table 1 are proper nouns, two of which refer to places of worship in the host context: المسجد الاخضر which means “the green mosque” and المسجد which means “the mosque.” These terms refer to different places in the Memphis area, and although these places have different names, مسجد النور and the “Memphis Islamic Center,” my wife and I made new names for these two mosques to accommodate Juman’s cognitive and linguistic ability. We used the word “green” to describe one mosque because of its interior green color and called the Memphis Islamic Center “the mosque” in Arabic because Juman’s linguistic skills were limited, and she was not able to articulate it in English. We employed a “the noun of the noun” construction to describe the first mosque and the format “the noun” to describe the second. A definite article indicated familiarity with the subject. As shown in Table 1, المسجد (“the mosque”) was the most common term that emerged in our family talks. This could be attributed to the fact that we regularly attended that mosque, which was also the one where Juman attended childcare in fall 2016 and spring 2017).

The Arabic words الكعبة السوداء ، مكة ، بيت الله (“the Black Kaaba, Makkah, House of Allah”) refer to the Kaaba, the partially black building at the center of the Great Mosque of Makkah, where Muslims around the world face when praying. The noun phraseبيت الله (“house of Allah”) is commonly used to describe this place, with the noun phrase describing the city where the Kaaba is located. However, my wife and I referred to the Kaaba with the noun phrase الكعبة السوداء (“the small black building”), which is not commonly used among Muslims. We coined this phrase during one of our visits because the black color of the Kaaba caught Juman’s attention. We kept using this phrase so that Juman could easily recall the place we were referring to.

Table 2 shows how الكعبة السوداء ، مكة ، بيت الله (“the Black Kaaba, Makkah, House of Allah”) was used in one conversation. This interaction shows referring expressions we used to construct the family’s religious identity while collaboratively socializing Juman.

In this excerpt, my wife and I were talking about the places we visited during our last vacation when we traveled to Saudi Arabia. My wife and I used referring expressions to talk about the Kaaba in order to construct our religious identity and connect it to a place we had recently visited. I included Juman in the conversation by asking if she remembered when we visited Makkah (Line 1). Juman responded by asking what Makkah was (Line 2). I clarified by using الكعبة السوداء (“black Kaaba”) because we used this term with Juman when we were there and بيت الله “House of God” because Salma usually used this term when talking to Juman about that visit. This conversation took place in our car and Juman was not fully following our talk. After a 7 s pause, I mentioned بيت الله (“House of God”), used plural attached pronouns رحنا (“that we”) twice in this talk, and tried to link the place to her grandparents, who we visited in Saudi Arabia during the same trip (Line 4). I tried to clarify more by adding that we saw a lot of people there, and Salma added that we saw a lot of children playing there too (Line 5). After a pause of 10 s, Juman tried to end the conversation by saying ماااا ادري (“I don’t know”) in a louder voice (Line 6). 

This interaction and use of linguistic devices showcased an instance of socialization because the effort at clarification and link to relatives (in this case, grandparents) created a supportive alignment among us as family members [28]. In addition, we were trying to direct Juman’s attention to the family religious heritage. However, Juman did not follow this conversation, possibly because it was taking place in the car, and she might not have been fully engaged in it. She was sitting in the back in her car seat playing with a toy, which she was looking at rather than at us or anything else.

One of the referring expressions emphasized in Excerpt 1 surfaced the day after the conversation in the car. This time, it was initiated by Juman. We were watching TV together, and some pictures of Makkah and the Great Mosque appeared. Juman initiated a conversation pointing at what she had seen (see Table 3).

As Juman saw the pictures on the TV screen in Table 3, she started saying, “Baba, Baba, look, look, House of Allah” with her voice going up (Line 1). She was trying to get my attention by addressing me as “baba, baba” with the verb شوف، شوف (“look, look”) and indicated what she wanted to say by saying بيت الله “House of God.” She might have used this rather than other terms highlighted in Excerpt 1 because it was easier for her to say. The Arabic term “the black building” was phonologically and syntactically more complicated as it has the voiced pharyngeal fricative /ʕ/, the glottal plosive /ʔ/, and double definiteness ال, which she had not acquired yet. The referring expression “Makkah” would be easier for her to produce, but she might have been unfamiliar with this term or what it refers to, as seen in Except 1, when she asked what Makkah was. In contrast, “House of God” in Arabic only contains two monosyllabic words—“house” and “Allah”—which were also familiar to her. 

In Line 2, I used reinforcement by saying, صح (“you’re right”) and used the same referring expression بيت الله (“House of God”) to indicate our mutual knowledge of the place she was referring to. Then, I linked the place to her grandparents with the word اللي رحنا (“who we visited”) in plural form. I believe I mentioned her grandparents for two reasons: 1) to make a stronger association with the place so Juman could remember it later, and 2) to build an attachment to the place by linking relatives to it. Juman responded to this statement by showing agreement and repeating the phrase بيت الله (“House of God”), as shown in Line 3. In this instance, I chose to accommodate her choice of wording and not mention the other referring expression used the day before. I repeated the referring expression she employed and reminded her of our visit by mentioning her grandparents in this talk in order to create a shared attachment and closeness to the Kaaba and build a sense of belonging. 

The other referring expressions المسجد (“the mosque”) and المسجد الأخضر (“the green mosque”) were also used in the family talk to indicate the places of worship where we would take Juman during weekends. The next talk in Table 4 was between Juman and me as I was asking her to get prepared to accompany me to the mosque.

In this excerpt, I asked Juman to put her shoes on, but she was not paying attention to what I said, so I repeated my request and mentioned the reason for going out was to go to prayer. We would use the verb نصلي (“pray”) to indicate the action of going out to pray (Line 2). Juman responded by asking how to pray (Line 3). Here, I understood that her question was not about the act of prayer but rather she was asking because she was not fully understanding my request as she was playing with her toys. In my response, I clarified where we were going rather than what we were going to do (Line 4). It can be seen in this line how first I mentioned المسجد (“the mosque”) and then followed by pointing out which one, المسجد الاخضر (“the green mosque”). Furthermore, I inserted the referring expression المسجد الأخضر (“the green mosque”) so she would understand the purpose of going out, and I mentioned who we were going to see عمو ر. (“Uncle R.”) so she could relate to the place we were going. Juman responded by saying يايالمسجد (“yay, mosque”) to indicate her excitement (Line 5). Then, I asked her again to put her shoes on after she understood my request. 

This excerpt illustrates how we linked places to relatives or friends when talking with Juman about our religious practices, such as when we linked our relatives in Saudi Arabia in Table 2 and Table 3 or the green mosque in Table 4. Here, the way I clarified my request by mentioning the place المسجد الأخضر (“the green mosque”) without explaining the act of prayer shows the connection we had with places before I tried to link the place to a family friend, Uncle R. In addition, this shows that we as parents did not aim to engage Juman in surface or deep discussion of religious acts at this age but rather sought to create a shared family identity by linking religious places to other families sharing the same background.

While my wife and I pursued a pro-bilingual FLP, Juman started going to an English-only childcare center, an environment that differed from the home culture and values. During this time, referring expressions containing a religious significance appeared in our daily interactions, revealing how we maintained and developed our religious identity and how Juman was engaged in this process. 

### 4.3. Frequent Use of Religious Phrases in Different Settings

The metalinguistic devices helped situate and discuss the family’s religious identity and people of the wider community. Therefore, throughout the pro-bilingual stage, when Salma and I chose to enroll Juman in an English-only childcare center, religious phrases were evident in our family talks and contributed to constructing our identity as an Arab Muslim family in the host country. During Juman’s enrollment at the center, my wife and I used basic religious phrases with Juman without explaining their deeper meanings. This is because Salma recommended not talking more deeply about religious concepts with Juman until the age of 7 because from Salma’s educational and religious point of view, the proper time to start religious education was between the ages of 7 and 10. However, I found that spontaneous use of religious phrases within family talk helped in constructing the shared family identity and socialize Juman into that identity. Table 5 shows the religious phrases that were evident in the recordings during this stage.

As shown in Table 5, الحمدلله (“praise be to Allah”/“praise God”)—which was usually used during meals and in certain other situations, such as after sneezing—was the most common religious phrase among all family members (Muhammad = 186, Salma = 155, Juman = 54). The second most common phrase ان شاء الله (“God willing”) usually referred to actions in the future and was more often uttered by Salma (*N* = 142) than me (*N* = 111) or Juman (*N* = 18). The third most common was بسم الله (“in the name of Allah”) in various contexts but mostly before meals (Muhammad = 90, Salma = 67, Juman = 3). Finally, ماشاء الله (“oh my goodness/oh my god”) was usually used to show astonishment and admiration (Salma = 83, Muhammad = 69, Juman = 11). As seen from the distribution, Juma used religious phrases far less than her parents, but their emergence in her turns when talking to family members indicated her active role in family talks involving religious phrases. Below, I give an example of the use of these religious phrases in our family talks and how they contributed to constructing the religious identity of our family.

Table 6 shows that I used two religious phrases in a conversation with Juman when I was trying to convince her to go to the childcare center, while she was insisting on going back to the Mother’s-Day-Out program at the mosque. In the excerpt, I used ان شاء الله (“God willing”) and ماشاء الله (“oh my goodness/oh my god”), showing how these phrases were employed in my talk with Juman to contribute to family identity construction and her linguistic and religious socialization.

While I was conversing back and forth with Juman about going to the childcare center, and after multiple attempts to portray the center as fun and interesting, I used the word مدرسة (“school”) in Arabic, but this was the word we constantly used to refer to any educational settings when we talked to Juman at this stage. I think this is because the word حضانة which is a closer equivalent to “childcare center” contains the /dˤ/ sound, which Juman was not able to produce yet. Then, I told her directly why it was important for her to go to the center and inserted the phrase *inshallah* (“God willing”) (Line 3). Juman interrupted my talk by repeating the same phrase she had been producing since the beginning of the conversation لا لا ما أبغئ (“no, no, that’s not what I want”). I completed my statement by inserting *inshallah* again in an attempt to convince her that the childcare center would help her use English, which is what she wanted. Again, Juman indicated her refusal by repeating the phrase ما أبغئ (“that’s not what I want”) twice. Then, I tried to tempt her again with the notion of speaking English and mentioned that people back home would be amazed by her ability to speak English. I used the phrase ماشاءالله (“oh my goodness/oh my god”), which is usually associated with amazement and admiration. However, Juman said she did not want to go there because she wanted to stay home with us (Line 7).

The excerpt also shows how I incorporated two religious phrases into my attempts to convince Juman to go to the childcare center. The first was encouraging Juman to go so she could speak English and make friends with the use of *inshallah* since this expression describes something that will hopefully happen in the future (God willing). The second was to entice her with the idea that people would say ماشاءالله(“oh my goodness/oh my god”) when marveling at her ability to speak English. Furthermore, the repetition of *inshallah* and the use of two religious phrases with Juman in this talk illustrates how repetition of certain phrases in family discourse with cultural or religious indexicality can contribute to identity maintenance or negotiating efforts.

This excerpt reveals examples of language and religious socialization in our family talks. Starting with language socialization, I intentionally told Juman that when we went back home, they would be amazed by her ability to speak English. This moment of socialization shows how the pressure from relatives surfaced in our talks with Juman. Salma and I heard so much about Juman’s English education from our relatives that it contributed to changing our language ideology and even appeared at the level of daily interaction.

In another conversation between Juman and Salma, Juman uttered the phrase الحمدلله (“praise be to Allah”) after sneezing although we had not instructed her to do that (see Table 7). This excerpt shows how Salma used praising strategies to highlight Juman’s use of religious phrases and socialize her into the value of good behavior. Furthermore, this excerpt shows Juman’s attempt to locate herself within the family identity construction.

In this excerpt, Salma used the adjective بطله (“you’re good”) after Juman said الحمدلله( “praise be to Allah”) and covered her nose and mouth when she sneezed (Line 1). This adjective is among several complimentary words and phrases Salma usually used when talking to Juman to praise her for good behavior or being clever. Juman asked her mother why she praised her (Line 2), and Salma explained her good behavior was covering her nose and saying *alhamdullilah*. Juman grasped that her mother valued her producing *alhamdullilah* and resumed the talk with her mother about this point. Thus, Juman showed her mother she knew the importance of the phrase by forming an interrogative sentence صح هو لازم نقول الحمدلله (“Is that right, we should say praise to Allah?”) (Line 4). Juman in this statement was not asking but rather showing her knowledge of the phrase and was situating herself in the family identity construction. Juman employed the verb نقول (“that we say”) with the prefix ن which includes the whole family not only her. Then Juman told her mother that she did not hear me saying *alhamdullilah* once as she used the past tense verb negation form بابا ما قال (“he didn’t say”) (Line 6). By stating this, Juman was trying to show her awareness of phrase usage and create closeness with Salma by accusing me of not using the phrase her mother liked. Salma laughed and asked her when this had happened. She said أول (“first”), indicating in the past and again repeated her statement that بابا ما قال الحمد لله (“Dad didn’t say ‘praise be to Allah’”). Juman at this age was using أول which is basically an ordinal noun to indicate the past, although we were not using this word to indicate tense. Salma again laughed and said, “He probably forgot.” Then Juman asked, in Line 10, “How did he forget?” Juman at this point was able to recognize the meaning and use of the verb ينسى which means she was asking because she wanted her mother to talk more about this phrase with her or just talk more in general. Salma was trying to wrap up the conversation with Juman by explaining briefly how I forgot by saying يمكن ما قالها(“maybe he didn’t say it”), and then she repeated her praising by saying انتي شاطرة (“you’re good”). 

This excerpt shows how the conversation started with Salma encouraging Juman for the proper use of a religious phrase and for covering her mouth when sneezing, but Juman understood that her mother was placing more value on the production of the religious phrase. This could be because we had already taught her to cover her nose and mouth when sneezing and this was something she usually did, so it must have been the religious phrase that got her mother’s attention and praise. Salma’s effort to praise Juman’s good language behavior and proper use of the religious phrase was thus a socialization moment [29]. Furthermore, Juman was enrolled in an English-only childcare center, where she was not socialized into saying certain linguistic phrases after sneezing. Instead, she was socialized into this act at home and used language to show her competence in this religious phrase and to engage in a social relationship with it. Juman’s participation in the talk and taking a stance by aligning with her mother showed how social relations were distributed.

Like Table 7, Table 8 illustrates how we as parents used praising techniques to socialize Juman into the value of using religious phrases properly and to construct a shared family religious identity through direct teaching. In this excerpt, we were all eating dinner together and Juman was about to leave the table. I asked her to put her dish in the sink, and then the conversation became about manners when eating and drinking in Islamic culture.

This excerpt shows first how I addressed Juman by name and asked her a question about what to say after finishing a meal (Line 1). In this attempt, I was trying to give Juman a space to be active in the process of conversing about Islamic culture. Although the religious phrase in this talk was similar to the one in the previous talk (Table 7), their association with actual practice was different. In this excerpt, I initiated the socialization about الحمدلله (“praise be to Allah”) once we finished eating, while in the previous excerpt, Juman initiated the socialization moment about the use of the same phrase after sneezing while conversing with her mother. Juman understood that I was asking about which phrase to use after eating but answered with the incorrect phrase for this context بسم الله (“in the name of Allah”) (Line 2). I changed the way I addressed Juman by saying حبيبتي (“darling”), which indicated I was trying to bring more closeness and attention in our talk and correct her by introducing the proper phrase to be said after eating الحمدلله (“praise be to Allah”) (Line 3). Here, I used the plural pronoun نقول (“we say”) in a declarative rather than imperative sentence with the singular pronoun أقول (“I say”) to indicate that we all shared this behavior and to establish a sense of familiarity with the context. Juman asked me again about الحمدلله (“praise be to Allah”) and it seemed she was aware of the pragmatic use of بسم الله (“in the name of Allah”) more than الحمدلله (“praise be to Allah”) and this question was her attempt to understand its usage. To answer her question, I tried to clarify by connecting the phrase to the proper time and action (Line 5). I explained that after eating and putting the dishes away, we say الحمدلله (“praise be to Allah.”) Again, I used the plural pronoun attached to all the verbs in this turn as in خلصنا/ رحنا/نقول (“that we say, that we go, that we finish”) in declarative sentences. Juman said طيب الحمدلله (“Okay, praise be to Allah”) to show she now understood when to say this phrase (Line 6). I then used praising strategies juxtaposed with the “darling” term of address. 

Finally, these excerpts demonstrate how the use of religious phrases in our family talks during the pro-bilingual FLP stage helped develop our family’s religious identity. In Excerpt 5, I used religious phrases to encourage Juman to go the childcare center so she could speak English. In Excerpt 6, Salma praised Juman for her proper use of a religious phrase when she sneezed. Juman recognized that we valued this cultural norm, so she tried to engage in talk with her mother about how I did not use this phrase after sneezing. Table 7 shows me teaching Juman dinner manners and using praising techniques to encourage her to use religious phrases. 

## 5. Discussion

This study revealed how identity was constructed and negotiated in a family through language socialization during a dynamic time for our FLP due to the family’s experience with mobility. The concentration on religious identity was due to the experience of Muslim parents raising their child in a predominantly non-Muslim context that differed from the home country context of Saudi Arabia and the family network of Muslim minorities in the U.S. The construction and negotiation of religious identity in our family developed along with Juman’s sense of bilingualism and biculturalism, supporting the notion in family discourse and identity research that individuals can create identity moment by moment through interactions employing linguistic devices [30]. The current study provided a glimpse into how religious identity was presented, constructed, and negotiated in a Muslim transnational family. I found two main strategies in our family talks contributing to the construction of our religious identity as a Muslim family. In keeping with how parents use language to socialize their children into the values and beliefs of the family and culture in daily interactions [31], family members use their linguistic resources to construct identities in interactions [28] and shape their FLP. The strategies we employed consisted of 1) using referring expressions to talk about religious places in Saudi and host contexts, and 2) frequently using religious phrases in different settings. 

In this study, religious referring terms were used frequently in the family to indicate the family’s religious identity and link religion to places of worship and locations in the home country. Religious referring expressions were also used to link people, such as when Juman asked about her grandparents when we talked about Makkah. At this stage of religious identity construction, Salma and I coined some of these referring terms to make ideas easier for Juman to understand and use. For instance, we created the term الكعبة السوداء (“the black box”) and بيت الله (“House of Allah”) to refer to Makkah, which Juman used when she initiated a talk to point out Makkah on the TV. Our parental effort to introduce religion through referring terms reflected our strong language ideology and attachment to Arabic and its associated Islamic culture. This is similar to how Arab parents in immigrant families value Arabic to maintain the family’s ethnolinguistic identity [21,32].

Another pattern in family talk was that all family members used religious phrases, which contributed to share identity construction and our parental efforts to socialize Juman into our cultural and societal norms. This finding echoes Moore’s studies [33,34], who found repetition practices among the Maroua Fulbe community to create a link to the Quran and become competent in religious practices, which resulted in children developing linguistic competence in Arabic, their L2. However, in our family, the frequent use of religious phrases helped socialize Juman into the social and cultural practices of the home culture, such as incidents where we discussed with Juman what to say before and after eating and how we frequently used praising techniques when she produced terms correctly. 

The study found that the frequent use of religious phrases—while the family was pursuing a bilingual FLP and Juman was attending an English-only childcare center—revealed the identity construction mechanism of a transnational Muslim family in the U.S. Employing established praising strategies such as بطله (“you are good”) and shifting between addressing names in one talk shows how we as parents socialized Juman into our values and cultural and religious manners. Parents in multilingual families tend to employ addressing terms to construct new cultural and social identities, as with the case of Chinese families in the UK [35], or to socialize children into a particular cultural norm, such as valuing hierarchy among Korean immigrant families in the U.S. [36,37]. In our case, we used praising strategies, such as بطله (“you’re good”) in Excerpts 5 and 6, to socialize Juman into our religious identity.

## 6. Conclusions

This study showed the development of religious identity in our family and how our child perceived and participated in that development. This study explored the practices transnational families of through exploring diverse experiences in dealing with multilingualism, that can be understood through parent language ideology, the enactment of FLP, and factors related to identity construction. The study showed how the family experiences affect the way individual family members perceive social relations and social structures and construct and present their own identities. The study indicated that children family’s transnational experience and the dynamic of FLP were affected not only family talk style but also through identity construction. The main finding of the study entailed the analysis of personal auto-ethnographic accounts that explored the emergence of religious identity in the family talks via a) using referring expressions to talk about religious places related to two several contexts, and b) frequent use of religious phrases in different settings, that revealed the interplay between macro and micro factors affecting the parental language ideology, language planning, and identity construction in our FLP.

This study contributed to the field of language policy and translanguaging family talk by presenting the different trajectories in identity construction and family language in bilingual and multilingual family’s contexts from less studied religious and ethnic groups. Since this study focused only on one family and due to the small size of the participants, future studies could examine the different trajectories in identity construction in other Arab transnational families and other types of families from different religious and ethnic backgrounds with large sample. While this study showed our child’s identity construction at an early stage, it would also be helpful to examine how young adults in transnational families perceive and participate in their family’s identity construction. Such studies would help to better understand language and religious socialization at different stages as well as the FLP process. Future studies could extend this line of research by including different religious minorities and exploring religious identity construction in multilingual families.

## Figures and Tables

**Table 1:** The percentage of referring expressions for religious places.

		Muhammad	Salma	Juman
المسجد الاخضر	the green mosque	26/220 (28.2%)	46/223 (20.7%)	13/109 (11.9%)
الكعبةالسوداء	the black Kaaba	7/220 (3.2%)	11/223 (4.9%)	2/109 (1.8)
بيت الله	House of Allah	32/220(14.5%)	19/223 (8.6%)	7/109 (6.4%)
مكة	Makkah	12/220 (5.4%)	9/223 (4%)	5/109 (4.6%)
المسجد	the mosque	107/220 (48.7)	138/223 (61.8%)	82/109 (75.3%)

**Table 2:** Excerpt 1.

Participants	The Participants Speech	Translation
Muhammad	تذكرين جوجو يوم رحنا مكة؟	You remember Jojo when we went to Makkah?
Juman	وش مكة؟ <creaky noise>	What’s Makkah? <creaky noise>
Muhammad	اقصد.. امممم بيت الله ، الكعبة السوداء	I mean house of Allah, the black box.
Muhammad	جمان، اللي ماما قالت لك هذا بيت الله، يوم رحنا عند جدة و جدة.. كان فيه ناس كثير:	When mama said this is house of Allah, when we went to grandparents, there were many people.
Salma	حبيبي جمان، كان فيه ناس كثير و كان فيه بنات و أولاد صغيرات يلعبون	My love, Juman, there were many people and there were many small kids playing there.
Juman	ادري/؟؟/ ::ما	I don’t know /؟؟/

**Table 3:** Excerpt 2.

Participants	The Participants Speech	Translation
Juman	بابا بابا:شوفشوف بيت الله؟؟	Baba Baba: look look, House of Allah?
Muhammad	صح حبيبي .. هذا بيت الله اللي رحنا مع جد و جدة	You’re right darling, this is the House of Allah we visited with your grandparents.
Juman	ايوه بيت الله	Yes, House of Allah.
Juman	بابا متى نروح عند بيت الله؟؟	Baba, when are we going to House of Allah??
Muhammad	ان شاء الله حبيبي نروح.. السنة الجاية	Inshallah darling, next year.
Juman	بس متى يا بابا؟!	But when, Baba?
Muhammad	اذا رحنا عند جد و جدة	When we go to see your grandparents.

**Table 4:** Excerpt 3.

Participants	The Participants Speech	Translation
Muhammad	جما::ن البسي شوزكبنطلع	Juman, put on your shoes, we’re going.
Muhammad	جما::نيالله، بنطلع نصلي	Juman, yallah, we’re going to pray.
Juman	كيف نصلي؟	How to pray?
Muhammad	يعني نروح المسجد ، المسجد الأخضر اللي عنده عمو ر.	I mean we are going to the mosque, the green mosque, where Uncle R. goes.
Juman	<singing>يا:ي المسجد<	<singing> Yay, the mosque>
Muhammad	طيب يالله البسي عشان نطلع<noise>	Yallah, put on your shoes so we can go <noise>

**Table 5:** Percentage of participants’ speech distribution of religious phrases.

Religious Phrases		Muhammad	Salma	Juman
الحمد لله	Praise God	186/456 (40.8%)	155/456 (34.7%)	54/101 (53.4%)
بسم الله	In the name of Allah	90/456 (19.7%)	67/456 (14.9%)	33/101 (32.8%)
ان شاء الله	God willing	111/456 (24.3%)	142/456 (31.8%)	3/101 (2.9%)
ما شاء الله	Oh my goodness	69/456 (15.2%)	83/456 (18.6%)	11/101 (10.9%)

**Table 6:** Excerpt 4.

Participants	The Participants Speech	Translation
Muhammad	لازم جماني تروحين المدرسة عشان..بس	But you have to go to the school, Juman, because
Juman	لا: لا: لا ما ابغى	No, no, no. that’s not what I want.
Muhammad	<*exhales*>عشان جمان حبيبي ان شاء الله تكبرين و تتكلمين انقليزي	<*exhales*> So you darling grow up and you can speak English
Juman	<*high-pitched*>ما:: ابغى ما:: ابغى	<*high-pitched*> That’s not what I want, that’s not what I want!
Muhammad	بعدين تروحين السعودية و يقولونماشاء الله تتكلم انقليزي؟	Then you’ll go to Saudi Arabia, and they’ll say, “oh my goodness, she speaks English.”?
Juman	<*extra high pitch*>لا لا ما ابغى	<*extra high pitch*> No, no! I don’t want to!
	((pause))	((pause))
Juman	ابغى اجلس معكم	I want to stay with you.

**Table 7:** Excerpt 5.

Participants	The Participants Speech	Translation
Salma	بطله جمان!	You’re good, Juman!
Juman	ايش ماما؟	What, mama?
Salma	انتي شاطره لانكغطيتي/ فمك/و قلتيالحمدلله	You’re good because you covered your mout/ and said “praise be to Allah.”
Juman	صح هو هو.. لازم نقول الحمدلله؟	Is that right, we should say “praise be to Allah”?
Salma	/???/	/???/
Juman	بس بابا ما قال الحمدلله	But dad didn’t say “praise be to Allah.”
Salma	*<laughs slightly>* متى	*<laughs slightly>* When?
Juman	هو اول ما قال الحمدلله	First! He didn’t say “praise be to Allah.”
Salma	*<laughs>*يمكن نسي<	*<laughs>* He probably forgot>
Juman	كيف نسي؟	How did he forget?
Salma	امممممم يعني ما قالها بس انتي شاطرة	Ummm, I mean he did not say it but you’re good.
	*Laughs>*	*<laughs>*

**Table 8:** Excerpt 6.

Participants	The Participants Speech	Translation
Muhammad	اذا خلصنا الأكل جما:ن،وشنقوووول؟	When we are done, Juman, with our eating, what do we say?
Juman	/أمم نقول/بسم الله	Umm, we say */by the name of Allah*/
Muhammad	أ أ.. لا حبيبي نقو::ل الحمدلله	Um, no darling, we say “praise be to Allah.”
Juman	والحمدلله؟ <*coughs*>	And praise be to Allah? <*coughs*>
Muhammad	اذا خلصنا الأكل و رحنا نشيل الصحون نقول الحمدلله	If we are done with the food and we are putting the dishes away, we say “praise be to Allah.”
Juman	/طيب/الحمدلله	/Okay/, praise be to Allah.
Muhammad	بطله حبيبي، شيلي الصحن معك	You’re good, darling! Take the dish with you.

## Data Availability

The data presented in this study are available on request.
